# Assessment of right ventricle in pulmonary arterial hypertension with three-dimensional echocardiography and cardiovascular magnetic resonance

**DOI:** 10.2459/JCM.0000000000001250

**Published:** 2021-09-03

**Authors:** Mariangela Lattanzio, Laura Scelsi, Michele Golino, Maddalena Lattuada, Claudia Raineri, Annalisa Turco, Chiara Giuntini, Francesca Ceriani, Marco Curti, Andrea Bonelli, Filippo Piacentino, Massimo Venturini, Sergio Ghiringhelli, Fabrizio Morandi, Roberto De Ponti, Stefano Ghio

**Affiliations:** a Department of Heart and Vessels, Ospedale di Circolo & Fondazione Macchi, University of Insubria, Varese; b Division of Cardiology, Fondazione IRCCS Policlinico San Matteo, Pavia; c Division of Cardiology, Città della Salute e della Scienza, Ospedale Molinette, Torino; d Department of Diagnostic and Interventional Radiology, Ospedale di Circolo, University of Insubria, Varese; e Division of Cardiology, Ospedali ‘Spedali Civili’, Brescia, Italy

**Keywords:** three-dimensional echocardiography, magnetic resonance imaging, pulmonary artery hypertension, right ventricle

## Abstract

To correlate 3-D Echo and CMR RV parameters and to verify whether they are similarly related to the clinical conditions of patients with pulmonary arterial hypertension (PAH), a disease in which the RV plays a crucial prognostic role.

**Methods:**

We enrolled 34 consecutive PAH patients followed by our PAH clinics. All patients underwent a 3-D Echo and CMR assessment of RV volumes and functions in the same day. The presence or absence of correlation between major findings was investigated; functional RV parameters were also analyzed in relation to 6-min walking test (6MWT) results and BNP/Nt-proBNP plasma levels. Twenty-four subjects served as controls.

**Results:**

Good agreement was found between 3-D Echo and CMR measures of RV volumes [RV-end-diastolic volume (*r* = 0.72, *P* < 0.0001), RV-end-systolic volume (ESV) (*r* = 0.80, *P* < 0.0001)] and function [RV-EF (*r* = 0.73, *P* < 0.0001), RV-ESV/SV (*r* = 0.83, *P* = 0.001)] for all the subjects of the study. These correlations were stronger in PAH patients than in control subjects. Importantly, 3-D Echo and CMR RV-EF and RV to pulmonary arterial coupling (RV-ESV/SV) similarly correlated with BNP/Nt-proBNP levels and with functional capacity measured at 6MWT in the PAH patients group.

**Conclusions:**

3-D Echo demonstrated a significant agreement with CMR in the assessment of RV volume and function in PAH patients. Both techniques showed a similar correlation with clinical and prognostic parameters. The use of 3-D Echo should be amply boosted in the real-world clinical evaluation of PAH patients.

## Introduction

There is a long-standing, widespread agreement on the prognostic relevance of right ventricular (RV) function in patients with pulmonary arterial hypertension (PAH).^1–3^ The progression of the disease and survival are the capability of the RV to adapt to the chronically elevated pulmonary artery pressure (PAP).^4^

However, since the 1980s, the indirect assessment of RV function based on hemodynamic parameters has remained the most important guide to the clinical management of such patients and international guidelines have not produced practical recommendations on how noninvasive imaging of RV morphology and function should be performed to stratify prognosis: in fact, current guideline recommendations are limited to the assessment of the right atrial area and the presence of pericardial effusion.^4^ A comprehensive pathophysiological approach combining simple echocardiographic indicators of the systolic function of the RV, of the degree of tricuspid regurgitation and systemic venous congestion, has recently proved useful to stratify the prognosis of PAH patients.^5,6^ However, it must be acknowledged that, due to the complex geometry of this cardiovascular chamber, the assessment of RV systolic function by echocardiography is limited.

Cardiac magnetic resonance (CMR) is considered the gold standard for the evaluation of volumes, mass, and ejection fraction (EF) of both ventricles.^7–12^ In addition, CMR allows, without the necessity for obtaining hemodynamic measures, the RV arterial coupling to be calculated, a clinically relevant surrogate of RV contractility.^13,14^ Technological advances in three-dimensional echocardiography (3-D Echo) have also recently made possible the quantification of volumes and function of the RV, but few comparisons between 3-D Echo and CMR have been performed in PAH patients and yielded discrepant results.^15–18^

This study aimed at exploring the correlations between volumetric and functional parameters of the RV obtained both with the 3-D Echo and the CMR in consecutive PAH patients; the secondary aim was verifying whether 3-D Echo or CMR parameters are similarly related to the clinical conditions and functional capacity of these patients.

## Methods

### Patients

From January 2019 to June 2019, we studied 34 consecutive patients with age >20 years old followed by the Pulmonary Artery Hypertension (PAH) clinics of the ‘Ospedale di Circolo & Fondazione Macchi’ in Varese, Italy (‘Center 1’) and of the ‘Fondazione IRCCS Policlinico San Matteo’ in Pavia, Italy (‘Center 2’). Demographic characteristics (age and sex), clinical data (BMI, BSA, NYHA class), classification of PAH and laboratory findings (BNP and NT-proBNP) were recorded, when available. Both walking endurance and aerobic capacity data were acquired from a 6-min walking test (6MWT), whereas hemodynamic data (such as mPAP, PCWP, SVR and CO) were derived from RHC; lastly, information about PAH-specific therapy and the number of drugs were also obtained (Table 1). All patients underwent echocardiography, including 3-D acquisition, and CMR on the same day. A group of consecutive patients admitted to the echocardiographic clinics of the same hospitals, who performed CMR for different reasons (i.e. suspected myocarditis, or pericardial or aortic disease) and had no evidence of structural heart disease, served as controls.

**Table 1:** Main clinical characteristics of PAH patients and controls

	PAH patients (*n* = 34)	Controls (*n* = 27)	*P*-value
Age (years)	61.8 ± 15.7	41.7 ± 15.7	<0.001
Gender (M/F)	13/21	15/12	0.18
BMI (kg/m^2^)	1.70 ± 0.18	1.83 ± 0.20	0.74
WHO class (I/II/III/IV/unknown)	2/21/9/0/2	–	–
BNP (*n*, pg/mL)^a^	150.70 ± 102.82	–	–
NT-proBNP (*n*, pg/mL)^b^	904.30 ± 1596.53	–	–
6MWT (m)^c^	406.25 ± 118.74	–	–
CI (L/min/m^2^)^d^	2.93 ± 1.04	–	–
PAPm (mmHg)^d^	39.20 ± 12.76	–	–
PAWP (mmHg)^d^	8.87 ± 2.58	–	–
PVR (UW)^d^	7.60 ± 3.67	–	–
PAH-specific monotherapy/dual therapy/triple therapy/none (*n*, %)^e^	3 (9%)/23 (70%)/4 (12%)/3 (9%)	–	–

6MWT, 6-min walking test; BMI, body mass index; BNP, B-type natriuretic peptide; CI, cardiac index; NT-proBNP, N-terminal pro B-type natriuretic peptide; PAH, pulmonary artery hypertension; PAPm, mean pulmonary artery pressure; PAWP, pulmonary artery wedge pressure; PVR, pulmonary vascular resistance; RAP, right atrial pressure; WHO, World Health Organization.

a Data are available for 13 patients only.

b Data are available for 18 patients only.

c Data are available for 32 patients only.

d Data are available for 31 patients only.

e Data are available for 33 patients only; patients with no therapy were those receiving calcium-antagonists. Patients with infusion devices were not included.

People with poor echocardiographic acoustic window, with the presence of ventricular defects or severe tricuspid regurgitation that could underestimate some measurements, were excluded. Other CMR-specific exclusion criteria were: the presence of any MR-incompatible implant (cardiac pacemakers or defibrillators), a wide waist circumference (>150 cm) or a weight greater than 130 kg, arrhythmias such as atrial fibrillation, frequent ventricular extrasystoles or ventricular tachycardia, claustrophobia, stage 4 and 5 CKD (eGFR < 30 mL/min).

The investigation conforms to the principles outlined in the Declaration of Helsinki. The Ethical Committees gave the approval for the analysis of the RELY registry (Ethical Committee of Ospedale di Circolo & Fondazione Macchi, Varese, prot. 29/2012, and Ethical Committee of Fondazione IRCCS Policlinico San Matteo, Pavia, prot. 44784/2011, date of approval: 15 December 2011). All patients signed an informed consent agreement approved by the Institutional Review Board of both centers for observational, nonpharmacological, nonsponsored studies, which complies with Italian legislation on privacy (Codex on the Privacy, D. Lgs. 30 giugno 2003, n. 196).

### Three-dimensional echocardiography

A complete transthoracic echocardiogram was performed with an ultrasound system Vivid E95 (General Electric (GE) Healthcare, Little Chalfont, United Kingdom), whereas the postprocessing analysis of data was conducted using the pc-software GE EchoPAC. Both echocardiographic acquisition and offline analysis were performed by two experienced cardiologists and echocardiographers; for each patient, the acquisition of the images was repeated several times (minimum three times, maximum eight times) to minimize acquisition errors and to enhance image quality. The analysis of 28 3-D Echo was performed by three different experienced cardiologists in order to calculate the inter-observer variability. Intra-observer variability was performed by the same three experienced cardiologists on offline data at different times.

From a practical point of view, after performing a full two-dimensional echocardiographic examination, the 3-D ultrasound examination of the RV was conducted through the transducer GE Healthcare 4V-D Probe Collector and two main phases. The first phase consisted of two different steps:

(1) Image acquisition was performed with the probe placed on the left hemithorax at the level of the fifth or sixth intercostal space, with the ultrasound beam directed toward the right shoulder and the transducer marker pointing toward the left side; in this way, an apical four-chamber view was obtained.
(2) With the focus shifted to the right ventricle (RV) and a widening of the ultrasound beam sector, a ‘multislice’ and ‘multibeat’ 3-D acquisition was performed with a frame rate of four frames per second.

Then there was the second phase performed offline in the absence of the patient, which began with the transfer of the images from the ultrasound system to a separate pc-workstation. All the images were processed using the GE EchoPAC software.

**Fig. 1:**
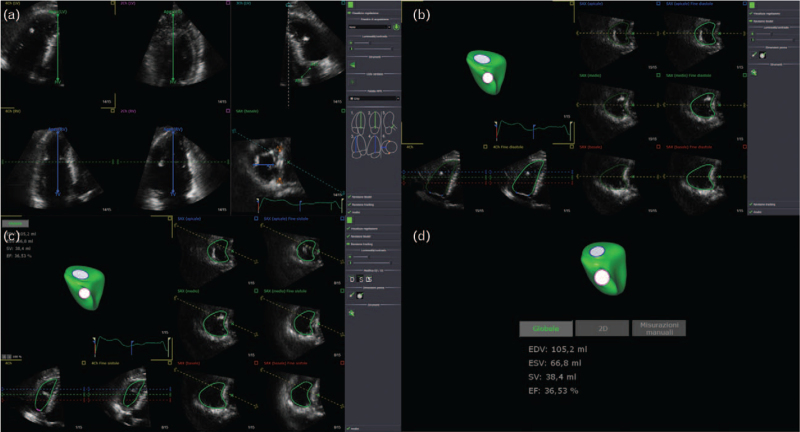
Phases of the postprocessing of echocardiographic images with GE EchoPAC software. (a) Finding landmarks with 4D RV function. (b) ‘Beutel revision phase’: the endocardial edge detection of the RV in the end-diastolic phase. (c) ‘Tracking revision phase’: the intracavitary perimeter was identified by the software during the entire cardiac cycle. (d) RV parameters and 3-D reconstruction. RV, right ventricle.

(1) In the apical four-chamber, three-chamber, two-chamber and short-axis view some landmarks necessary for the correct interpretation of the images by the software have been identified (in particular the tricuspid valve-apex distance and the mid-lateral distance of the RV) (Fig. 1a).
(2) Then there was the ‘Beutel revision phase’ consisting of endocardial edge detection of the RV in the end-diastolic phase by the software; since that process has not always been particularly accurate, a manual review of each measurement was performed (Fig. 1b).
(3) In the so-called ‘tracking revision phase’, the intracavitary perimeter was identified by the software during the entire cardiac cycle (Fig. 1c).
(4) Finally, based on the measurements made, the software quantified the volumes of the RV and produced a 3-D reconstruction of the chamber (Fig. 1d).

For each patient, the RV measurements provided by 3-D Echo were: end-diastolic volume (EDV, mL), end-systolic volume (ESV, mL), stroke volume (SV, mL) and EF (%).

Knowing the patient's weight, height and heart rate, it was also possible to index all the parameters for the BSA [EDV indexed (EDVi, mL/m^2^), ESV indexed (ESVi, mL/m^2^), indexed SV (SVi, mL/m^2^)] and to calculate cardiac output (CO, L/min) and cardiac index (CI, L/min/m^2^).

### Cardiac magnetic resonance

CMR was performed in both centers using a 1.5 Tesla Magnetic Resonance Imaging (Siemens Healthineers Magnetom Avanto-fit, Erlangen, Germany) with a phased array surface coil and ECG-gated sequences.

In all patients a noncontrast evaluation was performed; after the T2-based sequences, a steady-state free precession (SSFP) cine (end-diastolic) analysis was achieved of both ventricles.

**Fig. 2:**
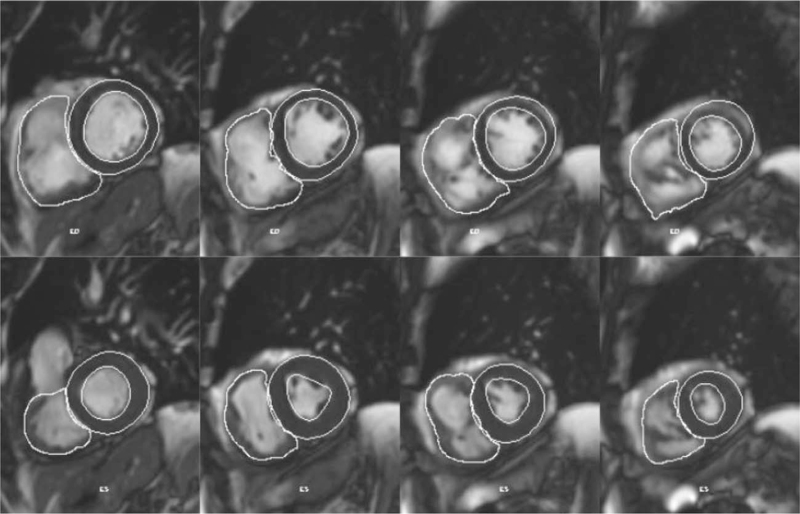
Semi-automatic determination of the RV endocardial and LV endocardial and epicardial edges in end-diastolic (upper line) and end-systolic phase (lower line) on contiguous short-axis sections (Siemens Argus software). RV, right ventricle.

All the images obtained were postprocessed and analyzed by an expert CMR radiologist on a pc-workstation Siemens Leonardo (Siemens Healthineers Magnetom Avanto-fit, Erlangen, Germany). The global biventricular function and volumes were determined following the manual identification of the left ventricle endocardial and epicardial edges and RV endocardial edge, both in the end-diastolic and end-systolic phase, on contiguous 8-mm-thick sections in short-axis view (Fig. 2).

The parameters provided by the software were: EDV (mL), ESV (mL), SV (mL), EF (%), CO (L/min), EDVi (mL/m^2^), ESVi (mL/m^2^), SVi (mL/m^2^), CI (L/min/m^2^).

### Statistical analysis

Continuous variables were expressed as mean and standard deviations while discrete variables were expressed as numbers and proportions. Regression analysis using the Least Squares Method was performed, considering a significant regression line slope if the *P* was <0.05. A Bland–Altman plot (difference plot) was used to analyze the agreement between 3-D Echo and CMR measurements in PAH patients and control subjects. Correlation analysis between imaging parameters and functional capacity was performed by Pearson's test (*r*) and coefficient of determination (*r* squared), considering significant values if >0.8.

All analyses were performed with the ‘STATA12’ software from Stata Corp LLC and MedCalc (version 12.5.0.0). Intra- and inter-observer variabilities were calculated by intra-class correlation coefficient *r* (Lin, 1999, 2000).

## Results

### Clinical characteristics of the subjects

Overall, 34 PAH patients and 27 controls were included in our study. No subject was excluded. Main baseline characteristics of the patients and control subjects (when available) are noted in Table 1. As expected, controls were younger than PAH patients (*P* < 0.001), without evidence of structural heart disease.

### Three-dimensional echocardiography and cardiac magnetic resonance findings

3-D Echo and CMR parameters are listed in Table 2. PAH-patients had lower values of RV-EF than controls, whereas RV-ESV/SV was numerically but not significantly higher in PAH than in control subjects with both techniques.

**Table 2:** Main 3-D Echo and CMR findings

	PAH patients (*n* = 34)	Controls (*n* = 27)	*P*-value
3-D Echo			
RV-EDV (mL)	89.67 ± 50.26	63.02 ± 22.17	0.21
RV-ESV (mL)	57.95 ± 41.94	28.24 ± 9.67	0.07
RV-EF (%)	38.55 ± 9.74	54.71 ± 6.87	0.03
RV-SV (mL)	31.51 ± 11.96	34.78 ± 14.32	0.44
RV-ESV/SV	1.78 ± 0.86	0.86 ± 0.31	0.35
CMR			
RV-EDV (mL)	156.83 ± 80.41	134.97 ± 37.93	0.49
RV-ESV (mL)	92.81 ± 69.70	56.67 ± 20.99	0.44
RV-EF (%)	44.38 ± 13.81	58.76 ± 6.69	0.03
RV-SV (mL)	63.99 ± 23.07	78.39 ± 19.32	0.17
RV-ESV/SV	1.51 ± 0.90	0.72 ± 0.20	0.29

3-D, three-dimensional; CMR, cardiovascular magnetic resonance; EDV, end-diastolic volume; EF, ejection fraction; ESV, end-systolic volume; PAH, pulmonary artery hypertension; RV, right ventricle; SD, standard deviation; SV, stroke volume.

**Fig. 3:**
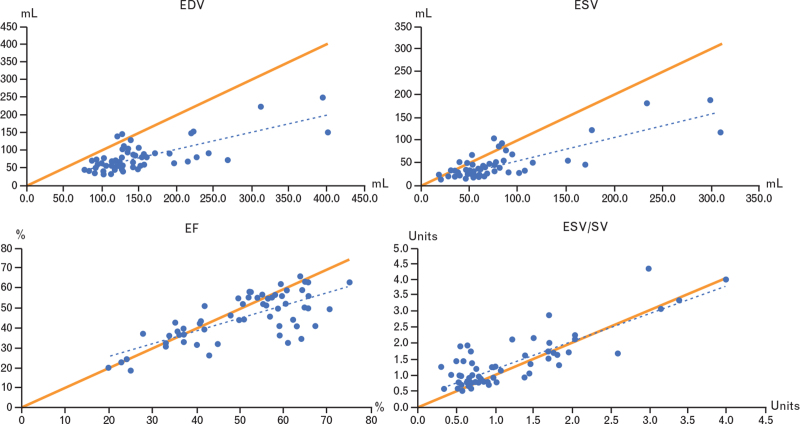
Linear regression (dotted blue line) and line of identity (solid orange line) comparing RV volumes (RV-EDV, RV-ESV) and function (RV-EF, RV-ESV/SV). EDV, end-diastolic volume; EF, ejection fraction; ESV, end-systolic volume; RV, right ventricle.

3-D Echo systematically underestimated RV volumes; the average difference for RV-EDV and RV-ESV was 69.6 and 32 mL, respectively. At regression analysis the data were not well distributed around the identity line (Fig. 3).

**Fig. 4:**
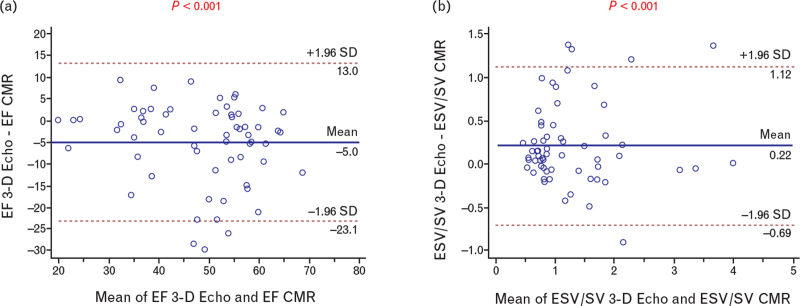
(a) Bland and Altman plot of differences between 3-D Echo and CMR vs. the mean of the two measurements for EF. Representation of limits of agreement (dotted lines) from −1.96 standard deviation to +1.96 standard deviation. (b) Bland and Altman plot of differences between 3-D Echo and CMR vs. the mean of the two measurements for ESV/SV. Representation of limits of agreement (dotted lines) from −1.96 standard deviation to +1.96 standard deviation. 3-D, three-dimensional; CMR, cardiovascular magnetic resonance; EF, ejection fraction; ESV, end-systolic volume.

Regarding RV function, the average difference for RV-EF and RV-ESV/SV in the entire population was 5% and 0.2, respectively (Table 2). At regression analysis the data were well distributed around the identity line (Fig. 3). These results did not differ when considering separately the two centers (Table S1). Besides, the Bland–Altman plots (Fig. 4a and b) did not show systematic differences between the two methods in the measurement of EF and ESV/SV.

Finally, Table 3 shows that the correlations between RV-EF and RV-ESV/SV with the distance covered during the 6MWT and with BNP or Nt-proBNP plasma levels (whichever were available) were low but similar, whichever technique was used.

**Table 3:** Correlations between 3-D Echo and CMR measures of RV function and 6MWT and BNP or Nt-proBNP plasma levels in PAH-patients

CMR/3-D Echo parameters	6MWT^a^ (*r/R*^2^)	BNP^b^ (*r/R*^2^)	Nt-proBNP^c^ (*r/R*^2^)
RV-EF (CMR)	0.07/0.005	0.42/0.17	0.42/0.17
RV-ESV/SV (CMR)	0.07/0.004	0.58/0.33	0.54/0.29
RV-EF (3-D Echo)	0.05/0.002	0.57/0.33	0.51/0.26
RV-ESV/SV (3-D Echo)	0.04/0.001	0.65/0.43	0.50/0.25

3-D, three-dimensional; 6MWT, 6-min walking test; BNP, B-type natriuretic peptide; CMR, cardiovascular magnetic resonance; EF, ejection fraction; ESV, end-systolic volume; Nt-proBNP, N-terminal pro B-type natriuretic peptide; PAH, pulmonary artery pressure; *r*, Pearson's correlation index; *R*^2^, coefficient of determination; RV, right ventricle; SV, stroke volume.

a Data are available for 32 patients only.

b Data are available for 13 patients only.

c Data are available for 18 patients only.

### Three-dimensional echocardiography intra- and inter-observer variability

**Fig. 5:**
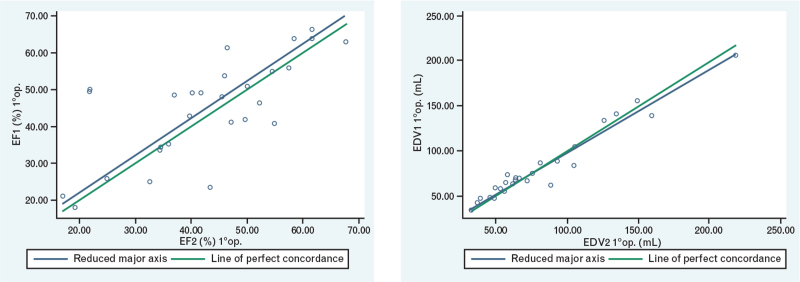
Correlation of the values measured by the first operator relating to the RV-EF and RV-EDV. EDV, end-diastolic volume; EF, ejection fraction; RV, right ventricle.

**Fig. 6:**
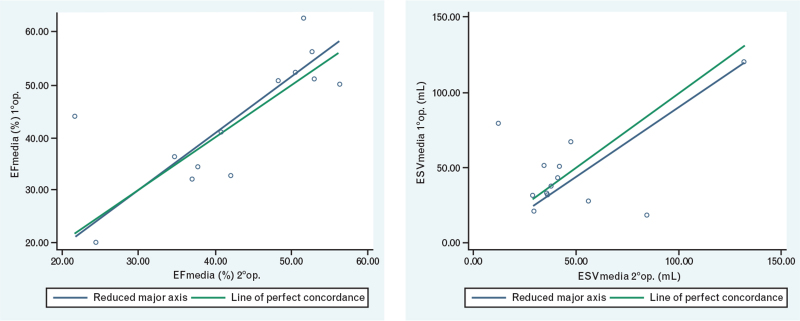
Correlation of the values measured by the first and second operators relating to the RV-EF and RV-ESV. EF, ejection fraction; ESV, end-systolic volume; RV, right ventricle.

All parameters exhibited good intra- and inter-observed correlation. Intra-observer variability was excellent for the three 3-D Echo measurements of RV-EDV and RV-ESV (respectively: *r* = 0.97, *P* < 0.001 and *r* = 0.98, *P* < 0.001); it was slightly lower for RV-SV (*r* = 0.86, *P* < 0.001) and for RV-EF (*r* = 0.83, *P* < 0.001) (Table 4 and Fig. 5). Inter-observer variability was good for RV-EF (*r* = 0.73, *P* < 0.001) and for RV-ESV (*r* = 0.67, *P* < 0.001), but not so for RV-SV (*r* = 0.28, *P* = 0.3) (Table 5 and Fig. 6).

**Table 4:** Intra-observer variability

3-D Echo measurement (first echocardiographer)	Mean ± SD	r	*p*-value
RV-EDV (mL)			
First	80.1 ± 40.6		
Second	79.9 ± 44.1	0.97	<0.001
Third	83.8 ± 44.2		
RV-ESV (mL)			
First	46.1 ± 33.5		
Second	46.1 ± 36.3	0.98	<0.001
Third	48.4 ± 38.1		
RV-SV (mL)			
First	34.0 ± 16.8		
Second	31.5 ± 15.4	0.86	<0.001
Third	34.6 ± 15.6		
RV-EF (%)			
First	44.9 ± 13.8		
Second	42.8 ± 13.6	0.83	<0.001
Third	44.2 ± 14.0		

3-D, three-dimensional; EDV, end-diastolic volume; EF, ejection fraction; ESV, end-systolic volume; r, intra-class correlation coefficient; RV, right ventricle; SV, stroke volume.

**Table 5:** Inter-observer variability: mean value of measurements of the first and the second echocardiographer with their correlation

3-D Echo parameters	First operator (m ± SD)	Second operator (m ± SD)	*r*	*P*-value
RV-EDV (mL)	81.2 ± 42.5	84.8 ± 36.2	0.48	0.041
RV-ESV (mL)	47.5 ± 36.1	50.2 ± 30	0.67	<0.001
RV-SV (mL)	33.4 ± 15.1	34 ± 15	0.28	0.301
RV-EF (%)	44 ± 13	42 ± 11.3	0.73	<0.001

3-D, three-dimensional; EDV, end-diastolic volume; EF, ejection fraction; ESV, end-systolic volume; r, intra-class correlation coefficient; RV, right ventricle; SV, stroke volume.

## Discussion

The main result of the present study is the finding of a good agreement between indices of RV function obtained at CMR and 3-D Echo, including not only RV-EF, but also the RV to pulmonary arterial coupling. Importantly, CMR and 3-D Echo parameters of RV function similarly correlated with BNP/NT-proBNP and with functional capacity of PAH patients, thus meaning that the two techniques may portend a similar clinical value in this disease.

The background to our study is that, despite extensive literature reporting that several echocardiographic variables may provide significant prognostic information in PAH patients, imaging of the right heart with 2-D Echo (due to the inherent limits) has not yet been considered as providing sufficient prognostic information to serve as an integral part of the treatment goal.^4^ Deriving and validating imaging biomarkers for an accurate evaluation of RV function is considered a major research priority to be addressed in the next 5 years.^19^ Although 3-D Echo is emerging as an interesting source of prognostically relevant data in PH, the few studies in the literature which have attempted to validate 3-D Echo in adult populations of PAH patients have reported contrasting results.^15–18^

In the present study, 3-D Echo substantially underestimated the ESV and EDVs of the RV. This result is consistent with data in the literature obtained in normal subjects and patients with congenital heart disease. Interestingly, the correlation between volume measurement with both techniques was weaker in the control group rather than in PAH patients. This can be explained if we hypothesize a greater precision in recognizing the endocardial edges of the RV at echocardiography when the RV chamber is dilated and thus the RV trabeculae are better identifiable.

As expected, the measurements performed by the same operator showed a linear correlation, very close to perfection. On the other hand, a certain discrepancy of measurements obtained by different operators (inter-observer variability) emerged, especially as regards SV, EDV and ESV but the inter-observer correlation of the RV-EF proved to be good.

The discrepancy in volume measurements can be explained by the poor resolution of the echocardiographic images of some patients, which makes difficult the tracing of endocardial edges, especially in the end-diastolic phase of the cardiac cycle. On the contrary, the accuracy between the two techniques was substantially greater in the assessment of RV functional measurements, RV-EF and RV to arterial coupling. This can be explained considering that discrepancies occurring both in end-diastole and in end-systole are eliminated when both are included in a ratio, as in the calculation of EF and of SV/ESV. Importantly, with the limit that the present population includes a relatively small number of subjects, the analysis shows that there is no systematic bias in the measurement of EF and ESV/SV with 3-D Echo as compared to CMR. To substantiate the clinical validity of 3-D Echo measurements is the observation that 3-D and CMR parameters similarly correlated with the distance walked at 6MWT and BNP/NT-proBNP plasma levels. The correlations were better with BNP/NT-proBNP plasma levels than with the distance walked at the 6MWT, most likely because exercise capacity does not depend only on RV function, but also on several other factors such as age, sex, physical deconditioning and comorbidities. Overall, these correlations were quite poor; however, the relation between 6MWT and clinical, hemodynamic, and neurohumoral parameters in PAH patients has never been reported to be very robust, even though the distance walked is a well known predictor of survival in PAH patients.^20^

The take-home message of the study may be clinically relevant because there is a widespread agreement in the literature that prognosis in PAH patients is strictly dependent on the function of the RV, but despite the convincing and long-standing evidence that CMR is the gold standard for morphological and functional assessment of the RV, CMR is not part of the routine clinical practice to date in patients with PAH. The lower costs and the ease of access to the examination make echocardiography the most widely used imaging technique in PAH patients in the real world.

### Limitations of the study

The small number of patients enrolled in this study is a limitation; however, it must be considered that PAH is a rare disease. Although there was a significant difference in age between PAH patients and controls, this difference should not impact the correlation between imaging of the RV with the two techniques. Finally, the study did not aim at identifying which index of RV function (RV-EF or RV-SV/ESV) is a better predictor of the clinical conditions or of the exercise capacity in PAH patients; the clinical value of each of these indices of RV function in PAH was out of the scope of the present study.

## Conclusion

3-D Echo demonstrated a good agreement with CMR in the assessment of RV volumes and in particular of RV function in PAH patients. Both techniques showed a similar correlation with other clinical prognostic parameters. The use of 3-D Echo should be amply boosted in the real-world clinical evaluation of PAH patients.

### Conflicts of interest

R.D.P. has received lecture fees from Biosense Webster and Biotronik and his Institution has received an educational grant from Medtronic, Biotronik, Boston Scientific, Biosense Webster, Abbott.

None for the other authors.

## Supplementary Material

Supplemental Digital Content
