# Supplementary material for: Assessment of right ventricle in pulmonary arterial hypertension with three-dimensional echocardiography and cardiovascular magnetic resonance
Source: J Cardiovasc Med (Hagerstown). 2021 Sep 3;22(12):929–36. doi: 10.2459/JCM.0000000000001250 (PMC10414158; doi:10.2459/JCM.0000000000001250)
Supplement: Supplemental Digital Content [file jcarm-22-929-s001.docx]

**Supplementary Material**

**TABLE**

**Table S1. Correlations between 3-D Echo and CMR measures of RV volumes and function**

**in all subjects (divided by center of origin).**

|  | **Center 1**  **r/R^2^** | **Center 2**  **r/R^2^** |
| --- | --- | --- |
| RV-EDV (mL) | 0.73/0.54 | 0.83/0.69 |
| RV-ESV (mL) | 0.83/0.70 | 0.89/0.80 |
| RV-EF (%) | 0.92/0.84 | 0.69/0.48 |
| RV-SV (mL) | 0.53/0.28 | 0.54/0.30 |
| RV-ESV/SV | 0.95/0.90 | 0.84/0.71 |
| *RV=right ventricle; EDV=end-diastolic volume; ESV=end-systolic volume; EF=ejection fraction; SV=stroke volume.*  *Center 1= Ospedale di Circolo & Fondazione Macchi - Varese, Italy.*  *Center 2= Fondazione IRCCS Policlinico San Matteo - Pavia, Italy.*  *r = correlation index; R^2^= coefficient of determination.* | | |
